# Flexible Endoscopy With Non-invasive Ventilation Enables Clinicians to Assess and Manage Infants With Severe Bronchopulmonary Dysplasia

**DOI:** 10.3389/fped.2022.837329

**Published:** 2022-04-19

**Authors:** Wen-Jue Soong, Pei-Chen Tsao, Chia-Feng Yang, Yu-Sheng Lee, Chien-Heng Lin, Chieh-Ho Chen

**Affiliations:** ^1^Division of Pediatric Pulmonology, Children’s Hospital, China Medical University, Taichung City, Taiwan; ^2^Department of Pediatrics, Taipei Veterans General Hospital, Taipei City, Taiwan; ^3^Department of Pediatrics, Tri-Service General Hospital, National Defense Medical Center, Taipei City, Taiwan; ^4^Department of Pediatrics, School of Medicine, National Yang-Ming Chiao Tung University, Taipei City, Taiwan

**Keywords:** bronchopulmonary dysplasia, flexible bronchoscopy, premature infant, noninvasive ventilation, Soong’s ventilation

## Abstract

**Objectives:**

The objectives of the study were to determine the efficacy of flexible endoscopy (FE) to assess the approachable aeroesophageal tract (AET) and subsequent changes in clinical management in infants with severe bronchopulmonary dysplasia (sBPD).

**Methods:**

This retrospective study investigated sBPD infants who received FE measurement from 2011 to 2020. FE was supported with non-invasive ventilation (FE-NIV) of pharyngeal oxygen with nose closure and abdominal compression without any mask or laryngeal mask airway. Data on AET lesions, changes in subsequent management, and FE therapeutic interventions were collected and analyzed.

**Results:**

Forty-two infants were enrolled in the study. Two thin scopes (1.8- and 2.6-mm outer diameter) were used. FE analysis revealed 129 AET lesions in 38 (90.5%) infants. Twenty-eight infants (66.7%) had more than one lesion. Thirty-five (83.3%) infants had 111 airway lesions where bronchial granulations (28, 25.2%), tracheomalacia (18, 16.2%), and bronchomalacia (15, 13.5%) were the main complications. Eighteen esophageal lesions were found in 15 (35.7%) infants. No significant FE-NIV complications were observed. The FE findings resulted in changes in management in all 38 infants. Thirty-six (85.7%) infants underwent altered respiratory care with pressure titrations (29, 45.3%), shortened suction depth (17, 26.6%), immediate extubation (8, 12.5%), changed insertion depth of endotracheal tube (7, 10.9%) and tracheostomy tube (3, 4.7%). Twenty-one (50%) infants had 50 pharmacotherapy changes, including added steroids, anti-reflux medicine, antibiotics, and stopped antibiotics. Eighteen (42.8%) infants received 37 therapeutic FE-NIV procedures, including 14 balloon dilatations, 13 laser-plasty, and 10 stent implantations. Seven (16.7%) infants underwent surgeries for four tracheostomies and three fundoplications.

**Conclusion:**

Flexible endoscopy with this non-invasive ventilation could be a safe and valuable technique for direct and dynamic visual measurement of AET, which is essential for subsequent medical decision making and management in infants with sBPD.

## Introduction

Successful management of severe bronchopulmonary dysplasia (sBPD) requires a multidisciplinary approach to optimize respiratory care, proper pharmacotherapy, appropriate interventions, and assessment of comorbidities (1). It is important to identify the lesions and have individualized management strategies in each infant with sBPD. Flexible endoscopy (FE) is a well-established gold standard tool for the direct visualization of dynamic anomalies in the aeroesophageal tract (AET) (2–4). Infants with sBPD typically require numerous invasive management, which include invasive positive pressure ventilation (PPV), multiple intubation and extubation of endotracheal tube (ET) and feeding tube, and airway suctioning during prolonged hospitalization (5, 6). As a result, these fragile infants are vulnerable to iatrogenic AET lesions such as subglottic stenosis, tracheomalacia (TM), carina malacia (CM), bronchomalacia (BM) (7–9), and gastroesophageal reflux disease (GERD) (10–12). Studies have reported that less than 5% of BPD patients had normal airway evaluations (13). Treatment strategies are available to prevent and manage AET lesions, which can improve their outcomes. Therefore, it is important to perform a detailed FE assessment of the AET to clearly recognize the lesions. However, the existing AET anomalies and compromised cardiopulmonary status remain challenging for invasive FE procedures and related adverse events.

“Pharyngeal oxygen with optional nose closure and abdomen compression (PhO_2_–NC–AC)” is a novel model of non-invasive ventilation (NIV), which can provide oxygenated PPV without using an artificial device such as an Ambu bag, face or nasal mask, laryngeal mask airway, ET, or ventilator. Previous studies (14–19) have demonstrated its efficacy in providing adequate oxygenation and ventilation during FE procedures of the AET in pediatric patients, even in a high-risk population. To our knowledge, there is no study that investigated the use of FE with NIV (FE-NIV) support to evaluate infants with sBPD.

This study also aimed to describe (1) the findings of FE-NIV measurement in AET lumens and (2) the resultant changes in the clinical management of infants with sBPD.

## Materials and Methods

This retrospective study investigated infants with sBPD who underwent FE-NIV evaluation of AET from January 2011 to December 2020 in a tertiary medical center, *Taipei Veterans General Hospital* in Taiwan. Written informed consent was obtained from the parents/legal guardians prior to the FE-NIV procedure. This was approved by the ethical committee review board of the hospital (IRB-TPEVGH No.: 2021-08-015BC).

### Patients Enrolled

From the criteria described by the National Institute of Child Health and Development (NICHD) (8), infants were diagnosed with sBPD if (1) born prior to 32 weeks postmenstrual age (PMA), and required supplemental oxygen for ≥28 days and ≥30% oxygen concentration or PPV at 36 weeks PMA; and (2) born between 32 and 37 weeks gestational age, and required supplemental oxygen for ≥28 days and ≥30% oxygen concentration or PPV at 56 days of life. Since the use of high-flow nasal cannula (HFNC) was not specified, infants born before 32 weeks PMA or born between 32 and 37 weeks PMA were defined as having sBPD if on >2 LPM with any inspired oxygen at 36 weeks PMA or 56 days of life, respectively. Failure of extubation was defined as the need for re-intubation within 48 h of extubation. Infants with congenital cardiovascular diseases were excluded from the study.

### Patient Preparation

All FE-NIV procedures were performed at the bedside of the neonatal or pediatric intensive care unit. Procedural sedation was administered with intravenous midazolam (0.1–0.2 mg/kg) with or without ketamine (1–2 mg/kg), and spontaneous breathing was maintained as much as possible. Topical anesthesia with 2% lidocaine solution (1–2 ml/kg) was instilled directly into the distal airways or nostrils, larynx, and trachea of infants with or without invasive airways (ET or tracheostomy tube). A syringe catheter, which is a trimmed suction catheter connected on a 5-ml syringe, was used to deliver the lidocaine solution with rigid laryngoscopy. Continuous monitoring of vital signs, including non-invasive blood pressure every 3 min, was done throughout the course of FE-NIV.

### Non-invasive Ventilation Support

Infants’ original respiratory support devices, including nasal cannula, nasal prongs, artificial airway, and ventilator, were all replaced with the following NIV of PhO_2_–NC–AC (13–17) support, which is briefly described below.

**FIGURE 1:**
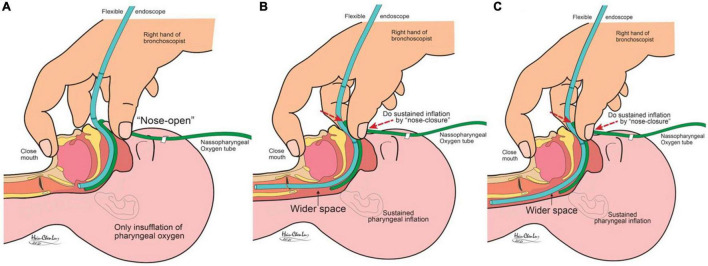
Illustrations show flexible endoscopy with non-invasive ventilation of “continuous nasopharyngeal oxygen with optional nose closure and abdomen compression.” **(A)** Nose open with narrow aeroesopharyngeal lumens; **(B)** sustained pharyngeal inflation with nose closure, which expands the lumens; and **(C)** for assessment, the scope tip may in pharyngeal, tracheobronchial, or esophageal lumens.

A warmed and humidified pure oxygen flow (1.0 L/kg/min) was continuously provided through a nasopharyngeal catheter (*via* the right nostril) to fill the upper airway space (Figure 1A). The infant’s mouth was closed by hooking the operator’s right index finger at the chin. The thumb and middle fingers were placed around the nose. The following procedures could be repeated to deliver the PPV (1). Inspiration was initiated by performing NC for 1–3 s with the thumb and middle finger. Cricoid pressure may be applied concurrently with the little finger (2). Expiration was started with the release of NC (Figure 1B). It could also be facilitated with the simultaneous use of AC over the umbilical region. These two steps were performed optionally at a rate of 3–5 cycles per minute. The operator could control both the FE and NC (release) maneuvers, while an assistant (if present) might provide the AC (release). If desaturation (<85% or 10% less than the prior baseline) or bradycardia (<100 beats/min) occurred for more than 30 s, the FE was removed, and NC-AC maneuvers were aggressively (5–10 cycles per minute) done.

### Flexible Endoscopy

The FE-NIV were proceeded through the following ways:

(a) For infants with invasive airways (ET or tracheostomy tube), FE was performed directly *via* the invasive airway to check the lumens inside and beyond the tip. Then removed the airways, while the tracheostomy wound was blocked with tape for the following FE-NIV examinations.
(b) For infants without invasive airway (or after extubation), FE-NIV was performed *via* the nostril (left) and examined down to all-approachable tracheobronchial lumens.
(c) Finally, the FE was withdrawn back into the pharynx, re-cannulated, and checked esophagus and stomach with proper insufflation of the esophageostomach by NC (Figure 1C).

After above FE-NIV procedures, the infants remained in the intensive care unit for continuous management.

### Data Collection and Analysis

Infants’ characteristics and information were collected from the electronic medical records. Data about the AET findings, related adverse events, and associated FE therapy were obtained from the FE reports. Airway malacia is defined as a lumen diameter reduction of more than 50% during spontaneous breathing. GERD is recognized as a visible, loose gastroesophageal junction, obvious reflux with mucosal erosion (20). Categorical variables were reported as frequency and percentage, while continuous variables were reported as median and interquartile range. All data analyses were performed using the Stata 13.1 software.

## Results

A total of 42 infants with a mean gestational age at birth of 27.1 weeks were enrolled in the study (Table 1). Thirty-two (76.2%) infants were transferred from other medical centers. At the time of FE, infants had a median PMA of 43.2 weeks, a median chronological age of 15.1 weeks, and a mean (SD) body weight of 3.4 (0.6) kg. Furthermore, 22 (52.4%) infants required respiratory support with invasive airway, 17 (40.4%) infants had ET, and 5 (11.9%) infants had tracheostomy tubes; PPV with nasal prongs was used in 16 (38.1%) infants, and oxygen cannulas were used in four (9.5%) infants. The leading indications for FE-NIV measurement were as follows: failure to wean PPV in 36 (85.7%) infants, failed ET extubation in 27 (64.3%) infants, and abnormal breathing sounds in 15 (35.7%) infants. Thirty-five (83.3%) infants had more than one indication.

**TABLE 1:** Characteristics of infants with severe bronchopulmonary dysplasia who received flexible endoscopy (FE) with novel^a^ non-invasive ventilation (NIV) support (*n* = 42).

Variable	Case number (%)
Gestation age at birth (weeks)^b^	27.1 (25.3, 29.5)
PMA^c^ at FE (weeks)^b^	43.2 (40.8, 48.2)
Chronological age at FE (weeks)^b^	15.1 (12.4, 19.5)
Body weight at FE (mean ± SD, Kg)	3.4 ± 0.6
Male sex	24 (57.1)
Respiratory supports prior to FE	
Endotracheal/tracheostomy tube	17/5 (52.4)
Nasal prongs-NIV	16 (38.1)
Oxygen	4 (9.5)
Indications of FE	
Failure to wean positive pressure support	36 (85.7)
Failed endotracheal tube extubation	27 (64.3)
Abnormal breathing sounds	15 (35.7)
Retractions	14 (33.3)
Suspected central airway problems	13 (28.6)
Persistent lung lesion	11 (28.6)
More than one indication	35 (83.3)

*^a^Pharyngeal oxygen (1.0 L/kg/min) with optional nose close and abdomen compression PhO_2_–NC–AC). ^b^Data are presented as median (interquartile range). ^c^PMA, post-menstrual age.*

### Endoscopes Used

Two thin flexible endoscopies without an inner channel, Olympus LFP scope (outer diameter 1.8 mm, working length 60 cm) or Olympus LFP scope (outer diameter 2.6 mm, working length 30 cm), were used for the FE-NIV procedures. Both were easily passed and assessed through 2.5- to 4.0-mm inner diameters of invasive airway, as well as from the nose to all approachable AET in infants without invasive airway. All FE-NIV were performed by authors WJ Soong and PC Tsao; both are qualified neonatologists, pulmonologists, and intensivists.

### Detected Lesions

A total of 129 AET lesions in 38 (90.5%) infants were identified (Table 2), which included 111 airway lesions in 35 (83.3%) infants and 18 esophageal problems in 15 (35.7%) infants. Among them, 28 (66.7%) infants presented with more than one lesion. Of the 111 airway lesions, the following were most frequently identified: bronchial granulation (28, 25.2%), TM (18, 16.2%), BM (15, 13.5%), and subglottic stenosis (11, 9.9%). Lobar bronchial granulations were right-side dominant (right 18, left 10, and bilateral 7), which might be caused by a deep-positioned ET and deep-suction trauma, resulting in local lumen narrowing or malacia. There were 18 esophageal lesions, which included 12 (66.7%) GERD, 3 (16.7%) esophageal inlet stenosis, and 3 (16.7%) mid-esophageal stenosis. Both sites of stenosis were defined as opening less than 3.0 mm with difficult FE (OD 2.6 mm) insertion.

**TABLE 2:** Findings and associated causes in aeroesophageal track using flexible endoscopy in infants (*n* = 42) with severe bronchopulmonary dysplasia.

Positive findings	Infants (%)	Lesions (%)	Associated causes^a^
Aeroesophageal tracts	38 (90.5)	129	
Airway	35 (83.3)	111 (100)	Iatrogenic
Laryngeal granulation		6 (5.4)	ET^b^
Subglottic stenosis		11 (9.9)	ET
Supra-stomy granulation		4 (3.6)	Stomy tube
Supra-stomy malacia^c^		5 (4.5)	
Tracheal granulation		8 (7.2)	ET/suction injury
Tracheomalacia		18 (16.2)	ET/PPV^d^
Carina granuloma		6 (5.4)	Deep ET/suction
Carina malacia		10 (9.0)	Deep/prolonged ET/PPV
Bronchial granulation		28 (25.2)	Deep ET/suction
Bronchomalacia		15 (13.5)	PPV
Esophagus	15 (35.7)	18 (100)	Iatrogenic
Inlet stenosis		3 (16.7)	Feeding tube
Mid-esophageal stenosis		3 (16.7)	EA^e^ anastomosis
GERD^f^		12 (66.7)	Labor breath
More than one finding	28 (66.7%)	75 (58.2)	Iatrogenic

*^a^Prolonged tissue damage. ^b^Endotracheal tube. ^c^More than 90% lumen collapse during quiet exhalation. ^d^Positive pressure ventilation. ^e^Esophageal atresia. ^f^Defined as visible loose gastroesophageal junction with reflux.*

### Adverse Events

The mean (±SD) duration of the FE-NIV procedure in infants with and without invasive airway were 102 (±33) s and 227 (±55) s, respectively (Table 3). Eight (19%) infants developed desaturation or bradycardia for more than 30 s, but all recovered within 60 s after aggressive NC-AC maneuvers. The duration of desaturation or bradycardia was less than 90 s. No pulmonary air leak related to the FE-NIV procedure was noted. All infants successfully completed the FE-NIV procedure.

**TABLE 3:** Procedural durations and complications associated with flexible endoscopy (FE) of the aeroesophageal tract in infants (*n* = 42) with severe bronchopulmonary dysplasia.

Items	Infant number (%)
Duration of FE procedure, seconds	
Via invasive airways,^a^ mean (SD)	102 (33)
Whole aeroesophageal tracts, mean (SD)	227 (55)
No significant complications	28 (66.7)
Desaturation (<85% between 31 and 90 s)	8 (19.0)
Bradycardia (<100 beats/min between 31 and 90 s)	4 (9.5)
Bleeding, mucosa	0
Pulmonary air-leak problem	0
Death	0

*^a^Endotracheal tube or tracheostomy tube.*

### Changes in Management

Flexible endoscopy findings led to 158 changes in subsequent management in 38 (90.5%) infants as shown in Table 4. There were 64 changes in respiratory care in 36 (85.7%) infants, which included PPV titrations (29, 45.3%), suction depth (17, 26.6%), tip position of artificial airway (10, 15.6%), and extubation (8, 12.5%). Twenty-one (50.0%) infants had changed 50 pharmacotherapies, which included add inhale or systemic steroids (15, 30.0%), anti-reflux medication (14, 28.0%) and antibiotics (8, 16.0%), and discontinued antibiotics (13, 26.0%) due to the found clean tracheobronchial lumens. In 18 (42.9%) infants, 37 therapeutic interventions were performed with FE-NIV, which included balloon dilatation (14, 37.8%) for narrow lumens, laser-plasty (13, 35.1%) for stenotic lesions or granulation ablations, and stent implantation (10, 27.0%) for severe (collapse of more than 90%) major tracheobronchial airway malacia that caused difficult weaning of PPV. Among these 10 stent implantations, there were four TMs, four CMs (two right and two left), and two left main BMs. Seven (16.7%) infants received surgical approaches due to failed medical management, which included four tracheostomies for severe subglottic stenosis and three fundoplications for severe GERD.

**TABLE 4:** Flexible endoscopy findings resulted in change of management of aeroesophageal track in infants (*n* = 42) with severe bronchopulmonary dysplasia.

Changes in management	Infant no. (%)	Change no. (%)	Rationales
**Involved**	38 (90.5)	158	
**Respiratory cares**	36 (85.7)	64 (100)	
PPV titrations		29 (45.3)	Moderate^a^ TM, CM, BM
Short depth of suctioning		17 (26.6)	Carina or bronchial traumatic granulations
Change depth of ET/tracheostomy tube		7/3 (15.6)	Avoids tip irritation at same site
Extubation of ET (to nasal prongs NIV^b^)		8 (12.5)	Acceptable central airways
**Medications**	21 (50.0)	50 (100)	
Add systemic steroids		15 (30.0)	Extubation, granulations
Add anti-reflux		14 (28.0)	Loose gastroesophageal junction
Stop antibiotics		13 (26.0)	Clear airways
Add antibiotics		8 (16.0)	Purulent secretion, preventive
**Therapeutic FE procedures**	18 (42.9)	37 (100)	
Balloon dilatation	9	14 (37.8)	Stenosis over larynx, trachea, and bronchi
Laser-plasty	7	13 (35.1)	Subglottic narrowing, granulations in trachea or bronchi
Stent implantation	6	10 (27.0)	Severe^c^ TM, CM, BM;
**Surgical procedures**	7 (16.7)	7 (100)	
Tracheostomy	4	4 (57.1)	Severe subglottic stenosis
Fundoplication	3	3 (42.9)	Severe GERD^d^

*BM, bronchomalacia; CM, carina malacia; ET, endotracheal tube; FE, flexible endoscopy; NIV, non-invasive ventilation; PPV, positive pressure ventilation; TM, tracheomalacia.*

*^a^Lumen collapse between 50 and 90%. ^b^Non-invasive ventilation. ^c^Lumen collapse more than 90%. ^d^Gastroesophageal reflux disorder.*

## Discussion

To the best of our knowledge, this is the first medical report wherein FE-NIV measurements were performed in the entire approachable AET in infants with sBPD. FE-NIV is a practical and well-tolerated technique, even in cardiopulmonary compromised populations. In this study, all infants had safely received complete, direct, and dynamic visual measurements within 7 min. Desaturation or bradycardia was transient and resolved with brief maneuvers of the NIV.

BPD is a clinically challenging condition. Many AET lesions, which result in fixed or dynamic lumen narrowing or incompetency, may be frequently overlooked due to poor diagnostic value of indirect radiographic images, incomplete measurement with artificial device block, or intolerant FE procedures without appropriate support. AET tissues of premature infants are particularly vulnerable to injury (21, 22). Since birth, they encounter frequent invasive trauma, such as intubations, suctions, infections, prolonged PPV, and systemic injuries of sepsis, hypoxia, and acidosis during their complicated and lengthy hospitalizations. These iatrogenic damages could result in AET tissue fibrosis, lumen narrowing, and structural weakness (23–25). Therefore, sBPD infants are best measured and managed by a multidisciplinary team that are specialized on comprehensive care of such diseases.

For infants with invasive airways, FE assessment through the artificial lumens should focus on the tissue around the tip of the tube, where most iatrogenic trauma happens. Manipulation of the artificial airway and FE can imitate the dynamic striking injury of airway tube and suction catheter, respectively, over the mucosa. These iatrogenic lesions may be ignored clinically. Therefore, a comprehensive measurement with FE-NIV should be performed from the nostrils to the whole approachable bronchi, esophagus, and stomach. Lesions around the pharynx, larynx, subglottis, and tracheostomy can be assessed (23–27). Kurachek et al. (28) reported that upper airway lesions like laryngomalacia, subglottic stenosis, etc., are the leading causes of extubation failure in pediatric patients. In our study, 38/42 (90.5%) of infants had AET lesions, which was higher than those in a previous report by Hysinger of only 20/27 (74.0%) infants having AET lesions (24). While, FE was assessed through the ET in 24/27 (88.9%) infants; therefore, only lesions beyond the distal tip of ET could be identified. Using the FE-NIV approach, we were able to precisely examine the whole AET and identify more lesions. These findings allowed further formulation of appropriate and individualized management strategies for respiratory care, pharmacotherapies, and invasive therapies. This highlights the importance of performing a complete and precise AET evaluation. During FE-NIV, the NIV can serve as a pneumatic stent and PPV, which eliminates airway resistance and respiratory work in infants with sBPD. Under direct visualization, the levels of lumen opening pressure, closing pressure, and PPV could also be titrated to ensure airway patency during subsequent respiratory care.

For symptomatic airway lesions such as granulations and stenosis, therapeutic interventions such as laser ablation or balloon dilatation could also utilize the FE-NIV (4, 16). For infants with severe TM, CM, or BM with frequent life-threatening episodes and failed response to conservative management, FE-NIV-aided stent placement (4, 17, 29) could avert more invasive surgical approaches such as tracheostomy, tracheoplasty, tracheopexy, or aortopexy. These FE-NIV interventions were all less invasive, safe, and well tolerated. In this study, tracheostomy and gastric fundoplication were reserved for severe and refractory subglottic stenosis and GERD, respectively.

This FE-NIV measurement of infants with sBPD provides several advantages. (1) Its practice is easy and simple, which obviates the need for artificial airway devices such as face masks, ventilation bags, laryngeal airway masks, ET, and ventilators. Compared with the HFNC, it may be better as less oxygen flow is needed, enough, and controllable PPV for assist and resuscitative ventilation even in an apneic status. (2) It is cost effective and applicable in resource-limited situations. (3) The PPV interface is located near the larynx with less ventilation dead space. Physiologically, it is similar to “apneic oxygenation (30, 31),” which can prolong the duration of safe oxygen saturation. (4) No existence of artificial device that allows extensive and unimpeded FE measurement; therefore, AET lesions could be identified more completely. (5) It can provide direct and dynamic visual evidence-based data to formulate individual clinical decisions and management. (6) It can safely provide detailed FE as well as more invasive therapeutic procedures (4, 15–18, 26) even in cardiopulmonary compromised infants.

This study had several limitations. (1) Due to its retrospective nature, it was difficult to clarify the complex factors contributing to these pathological lesions, especially for infants who were transferred to our hospital relatively late. (2) For FE-NIV, there was no control group against the traditional approaches of flexible bronchoscopy. (3) Bronchoalveolar lavage was not performed or included because that could easily be obtained with direct suction from the invasive airway. Hence, we preferred to use FE without an inner channel. (4) Although the technique of FE-NIV has previously been reported (14–19), it may still not be familiar to many pediatric pulmonologists. A future bronchoscopy training program with repetitive practice that makes it more translatable may be necessary. (5) The sample size was small and the FE-NIV used in the study was single-center based. Further multicenter studies of FE-NIV measurement in the whole AET may be necessitated.

FE-NIV could be a simple, safe, and valuable technique for obtaining direct visual and dynamic AET lesions in infants with sBPD. With this, subsequent medical decision making and individualized management may be optimized and advised accordingly.

## Data Availability Statement

The raw data supporting the conclusions of this article will be made available by the authors, without undue reservation.

## Ethics Statement

The studies involving human participants were reviewed and approved by IRB-TPEVGH No.: 2021-08-015BC. Written informed consent to participate in this study was provided by the participants’ legal guardian/next of kin.

## Author Contributions

W-JS: conceptualization, methodology, investigation, resources, writing-original draft, and supervision. P-CT, C-FY, Y-SL, and C-HL: investigation and project administration. C-HC: investigation and writing-review and editing. All authors contributed to manuscript revision, read, and approved the submitted version.

## Conflict of Interest

The authors declare that the research was conducted in the absence of any commercial or financial relationships that could be construed as a potential conflict of interest.

## Publisher’s Note

All claims expressed in this article are solely those of the authors and do not necessarily represent those of their affiliated organizations, or those of the publisher, the editors and the reviewers. Any product that may be evaluated in this article, or claim that may be made by its manufacturer, is not guaranteed or endorsed by the publisher.
